# The Area Law of Molecular Entropy: Moving beyond Harmonic Approximation

**DOI:** 10.3390/e26080688

**Published:** 2024-08-14

**Authors:** Amitava Roy, Tibra Ali, Vishwesh Venkatraman

**Affiliations:** 1 Department of Biomedical and Pharmaceutical Sciences, University of Montana, Missoula, MT 59812, USA; amitava.roy@umontana.edu; 2 Department of Mathematics and Natural Sciences, School of Data and Science, BRAC University, Dhaka 1212, Bangladesh; tibra.ali@bracu.ac.bd; 3 Department of Chemistry, Norwegian University of Science and Technology (NTNU), 7491 Trondheim, Norway

**Keywords:** entropy, area law, molecule, virtual screening, gas phase entropy, RRHO, harmonic oscillator

## Abstract

This article shows that the gas-phase entropy of molecules is proportional to the area of the molecules, with corrections for the different curvatures of the molecular surface. The ability to estimate gas-phase entropy by the area law also allows us to calculate molecular entropy faster and more accurately than currently popular methods of estimating molecular entropy with harmonic oscillator approximation. The speed and accuracy of our method will open up new possibilities for the explicit inclusion of entropy in various computational biology methods.

## 1. Introduction

Free energy governs all chemical processes. Change in free energy in a chemical process can be written as $\Delta G=\Delta H-T\Delta S_{\mathrm{th}}$, where *G* is the Gibbs free energy, *H* is the enthalpy, and $S_{\mathrm{th}}$ is the thermodynamic entropy governed by the second law of thermodynamics.

The second law of thermodynamics, as defined by Clasius [1], can be stated as follows: without outside intervention, heat flows from hot to cold as a non-equilibrium system reaches equilibrium. Ludwig Boltzmann connected the macroscopic definition of entropy from Clausius’s definition with the microscopic property of a system by the celebrated equation

(1) $$S_{\mathrm{th}}\left(E,N,V\right)=k_{B}\ln\Omega (E,N,V)$$

where $k_{B}$ is the Boltzmann constant and Ω is the total number of distinct microstates accessible to the system with the given macroscopic constraints. From the postulation that a thermodynamic equilibrium for an isolated system is the state of maximum entropy, Gibbs arrived at a similar form for the entropy of a system. However, the two methods differ in calculating the number of microstates and can provide different entropy values for the same system [2]. Following Gibbs’ formulation, thermodynamical entropy (also often referred to as Boltzmann–Gibbs entropy) can also be expressed by

(2) $$S_{\mathrm{BG}}=-k_{B}\sum_{i=1}^{n}p_{i}\;\ln\;p_{i},$$

where $p_{i}$ is the probability for the microstate *i* and *n* is the number of microstates. Equation (2) is similar in form to Shannon’s formulation of entropy of information [3],

(3) $$S_{\inf}=-\sum_{i=1}^{n}p_{i}\;\ln\;p_{i}$$

where $p_{i}$ is the probability of the event *i*, and *n* is the number of events. However, one must be careful to interpret any term defined by Equation (3) as thermodynamic entropy. Such an association has to explain the experimental observable of the heat flow from a hot to a cold body determined by the second law of thermodynamics.

The most noted work in connecting information-theory entropy with thermodynamic entropy was by ET Jaynes in his seminal paper “Information theory and statistical mechanics” [4]. Jaynes has argued that for a given macroscopic constraint, the randomness of the microstates can be estimated by information theory in the least biased way and staying maximally noncommittal about the system’s missing information (MI). Furthermore, the maximum-entropy principle is sufficient to develop the rules of statistical mechanics, from which the equilibrium thermodynamics properties can be calculated [4]. Jaynes’s maximum-entropy principle states that given precisely stated prior data, the probability distribution with the largest entropy best represents the current state of knowledge about a system [5].

### 1.1. Computational Techniques to Calculate the Entropy of a Single Molecule

Calculating free energy, which requires calculating the enthalpy and entropy, is required to predict the products of a chemical reaction. Change in entropy plays an essential role in some reactions. For example, large entropy changes can happen in the folding of biomolecules, ligand-binding processes, and the desorption of molecules from crystalline surfaces. An atomic-scale understanding that underlies these reactions can help efficient modeling of chemical reactions to predict their products. The accurate experimental measurement of entropy in solution is not trivial. However, gas-phase chemical dynamics has developed superb experimental methods to probe the detailed outcome of gas-phase chemical reactions [6]. These experiments help to benchmark the modeling of entropy from the first principles.

Temperature is not well defined for a single molecule; consequently, the entropy of a single molecule cannot be understood in terms of heat flowing from a hot body to a cold one. Instead, we will use a definition of entropy similar to the one used by Jaynes—entropy represents uncertainty in our current state of knowledge or missing information (MI) of a system under given macroscopic constraints. This article will refer to the entropy of a single molecule as $S_{\mathrm{mol}}$.

The most common approach for calculating the entropy of a single molecule is to approximate the dynamics of the molecule using Born—Oppenheimer approximation—the motion of an isolated molecule can be approximated by the motion of the nuclei in the potential created by the surrounding electrons. The approximation allows us to write molecular entropy as a sum of positional, orientational, and vibrational entropy, assuming that a molecule’s positional and orientational entropies are not coupled with the vibrational entropy of the molecule. However, orientational entropy is not necessarily decoupled from vibrational entropy for a flexible molecule [7]. Note that positional and orientational entropies are also referred to as translational and rotational entropies.

(4) $$S_{\mathrm{mol}}=S_{\mathrm{pos}}+S_{\mathrm{orie}}+S_{\mathrm{vib}}$$

Vibrational entropy can be divided into harmonic, anharmonic, and configurational entropies ($S_{\mathrm{conf}}$), especially for flexible and complicated molecules.

(5) $$\begin{array}{cc} S_{\mathrm{mol}} & =S_{\mathrm{pos}}+S_{\mathrm{orie}}+S_{\mathrm{HO}}+S_{\mathrm{anharm}}+S_{\mathrm{conf}}\\ \; & =S_{\mathrm{RRHO}}+S_{\mathrm{anharm}}+S_{\mathrm{conf}} \end{array}$$

$S_{\mathrm{HO}}$ denotes the entropy of a harmonic oscillator, $S_{\mathrm{anharm}}$ is the anharmonic correction, and $S_{\mathrm{conf}}$ is the configurational entropy that arises when a molecule can exist in multiple stable conformations at a given macroscopic constraint. Different conformations’ vibrational entropy must be appropriately averaged for a molecule with multiple stable conformations to calculate configurational entropy [8]. In practice, it is heuristically assumed that there is a single conformation with a residual entropy associated with the vibrational degrees of freedom, which is included in the $S_{\mathrm{conf}}$ [8]. Note that, in the literature, the terms configurational and conformational entropies are sometimes used interchangeably. Explicit calculations of $S_{\mathrm{anharm}}$ are often neglected or absorbed in the calculation of $S_{\mathrm{conf}}$. Calculating $S_{\mathrm{conf}}$ is intrinsically computationally expensive, as it requires, in principle, sampling the entire phase space. Consequently, calculating accurate entropy for a molecule can take thousands of CPU hours using molecular dynamics with molecular mechanics approximation (approximating the quantum mechanical interactions with a classical mechanical model [9]). Please see [10] for a recent effort to calculate $S_{\mathrm{conf}}$ for small molecules using molecular dynamics simulations.

One way to reduce computational resources is to approximate a molecule as a collection of simple harmonic oscillators (SHM), use a normal mode analysis technique (NMA), and calculate vibrational entropy. This approximation allows for avoiding identifying and counting microstates. The density matrix of a collection of SHMs is generally simpler to form in the energy eigenstates, eigenstates of the Hamiltonian, based on which entropy can be calculated, while avoiding identifying and counting microstates. However, this approximation of expressing the vibration of molecules as a collection of harmonic oscillators is not appropriate, as anharmonic vibrations play an equally important role in the dynamics of small molecules, as do harmonic vibrations [11]. Still, NMA calculations help examine a molecule’s dynamics [12]. Vibrational entropy is replaced by configurational entropy when a molecule can exist in multiple stable conformations at a given macroscopic constraint.

Calculating molecular entropy requires the calculation of orientational and positional entropy along with vibrational or configurational entropy. We can easily calculate molecules’ orientational and positional entropy from their geometry if we approximate them as rigid bodies. Please see [13] for a review of the topic. The entropy approximated by the rigid body and SHM approximation is referred to as $S_{RRHO}$ (Equation (5))—rigid rotor and harmonic oscillator approximation. To calculate the entropy of a molecule using SHM approximation, the charge distribution and the molecule’s geometry must be known. If the charge distribution is calculated using an approximation of the molecular mechanics, the entropy can be computed using CPU minutes to CPU hours of computational resources. If the charge distribution is calculated using quantum mechanical methods, it may take CPU days to CPU weeks of computational resources.

In this article, we propose an alternate way of calculating molecular entropy calculated from the surface properties of a molecule. The foundation of our approach is motivated by the area law of entropy.

### 1.2. Area Law—An Alternate Way of Calculating Entropy

Bekenstein, in his celebrated paper, proposed that the thermodynamical entropy of a black hole is proportional to its surface area [14]. The proposal is counter-intuitive as one would expect that the volume of the matter contains information; consequently, entropy should scale with volume but not surface area. In the article, Bekenstein explains how the counter-intuitive proposal is grounded in a deeper consideration of physics. At the same time, Hawking also derived a simple formula for a black hole’s entropy, equivalent to Bekenstein’s equation for entropy [15]

(6) $$S_{BH}=\frac{k_{B}A}{4L_{P}^{2}}$$

where *A* is the area of the horizon of the black hole, $k_{B}$ is the Boltzmann constant, and $L_{P}$ is the Planck length, the minimal value of the length used in the derivation (the ultraviolet cutoff $L_{UV}$). Hawking also showed that the area of the horizon of a classical black hole never decreases. As a consequence, the $S_{BH}$ of a classical black hole never decreases. The postulate that the total horizon area of classical black holes cannot decrease was recently proven experimentally by the Laser Interferometer Gravitational-wave Observatory (LIGO) [16]. The intriguing connection of black hole entropy with its surface area gave rise to several hypotheses on whether black hole entropy counts the microstate entropy and whether the relationship between entropy and area is a fundamental aspect of nature. For a review of the field, please check the reference [17]. Early seminal works identified quantum entanglement entropy as also proportional to area [18,19]. (See Supporting Information for a brief introduction to quantum entropy and quantum entanglement entropy.) Srednicki, in his work, showed that if a system of coupled harmonic oscillators is divided into two regions, oscillators inside an imaginary sphere *I* and oscillators outside the sphere *O*, and the density matrix is traced over the *O* oscillators, the entanglement entropy of the reduced density matrix is proportional to the area of the sphere that encloses *O* [19].

(7) $$S_{\mathrm{entanglement}}∼\mathrm{Area}.$$

For the derivation, Srednicki chose a local Hamiltonian, i.e., the entanglement between the oscillators, which lie far from each other, contributes very little to the entanglement entropy. Intuitively, for a quantum system with many microscopic degrees of freedom, the significant contribution to the bipartite entanglement entropy comes from the entanglement of the states of the degrees of freedom that lie near the boundary.

It is impossible to probe the area law of entanglement entropy with current experimental advancement. Reference [20] provides a detailed simulation study of superfluid ^4^He, where the area law of entropy was verified directly.

### 1.3. Thermal Entropy of a Single Molecule and Area Law

In the Boltzmann–Gibbs formulation, $S_{BG}$ is *additive*, i.e., $S_{BG}\left(A,B\right)=S_{BG}\left(A\right)+S_{BG}\left(B\right)$, where *A* and *B* are two independent subsystems. The subtle concept of *additive* in $S_{BG}$ leads to the *extensivity* property of classical thermodynamics; namely, the thermal entropy is proportional to the number of elements of the system when the number is large [21]. Consequently, the entropy varies with the volume.

The *additive* assumption does not hold for the entropy of one molecule. Different parts of the molecules can be correlated, and $S_{BG}$ may not be *additive* or *extensive*. For such cases, the idea of entropy has to be developed with *nonadditivity* as a central feature [21,22,23,24]. Surprisingly, *nonadditive* entropy is proportional to the area law, thus reconciling the area law in quantum systems with classical thermodynamics [21]. Motivated by this connection, we propose a method to estimate the thermodynamical entropy of a molecule from its surface property, as described below.

### 1.4. Estimating Molecular Entropy Using the Area Law

Embracing the area law of entropy from other fields of physics, we postulate that the thermodynamic entropy; i.e., given macroscopic constraints like pressure, volume, temperature, and number of atoms, the uncertainty in the microstates of a molecule is proportional to its surface area. The area law developed for the different systems, from black holes to simple harmonic oscillators to ^4^He ions, is for primarily spherical surfaces and occasionally for other regular geometric surfaces. Most molecular surfaces are not of any regular geometric shapes and contain various degrees of curvature, which can be measured by a molecular surface’s shape index (S) (see Section 2)) values. The deformations, i.e., deviation of shape index value from 1, arise when the surfaces of more than one atom overlap. Such deformations indicate atomic bonds. As the number of bonds in a molecule increases, the correlation in motion between different atoms in a molecule increases, and the uncertainty of our knowledge about the system’s microstates decreases. As the effect of the curvature in the area law of entropy has yet to be studied in detail, we make some assumptions for simplicity.

1. Surface deformations, curvature with an S value other than 1, add to the MI of the system’s microstates. Note that S value 1 indicates a perfectly spherical surface.
2. Positive (0 ≤ S < 1) and negative (−1 ≤ S < 0) deformations change the MI of the system independently.

We can express the MI about the molecular system due to the deformations with Shannon’s formulation of entropy of information (Equation (3)). Furthermore, following Jayne’s work [4], we can express thermodynamic entropy from the MI of the system. Consequently, we can write the thermodynamic entropy of a molecule ($S_{\mathrm{mol}}$) as a function of the area of the molecule, which we will refer to as $S_{\mathrm{th}}^{\mathrm{area}}$.

(8) $$\begin{array}{c} S_{\mathrm{mol}}=S_{\mathrm{th}}^{\mathrm{area}}\propto A_{\mathrm{area}\;\mathrm{of}\;\mathrm{the}\;\mathrm{molecule}\;\mathrm{with}\;S=1}\\ S_{\mathrm{th}}^{\mathrm{area}}\propto p^{+}\log\left(p^{+}\right)A_{\mathrm{area}\;\mathrm{of}\;\mathrm{the}\;\mathrm{molecule}\;0\;\leq \;S\text{<}1}\\ S_{\mathrm{th}}^{\mathrm{area}}\propto p^{-}\log\left(p^{-}\right)A_{\mathrm{area}\;\mathrm{of}\;\mathrm{the}\;\mathrm{molecule}\;S\text{<}0} \end{array}$$

Combining and incorporating the practice of dividing a surface into multiple smaller patches to carry out such calculations, we can write

(9) $$S_{\mathrm{th}}^{\mathrm{area}}=S_{0}+\sum_{i}\left[a+b\;p_{i}^{+}\log\left(p_{i}^{+}\right)+c\;p_{i}^{-}\log\left(p_{i}^{-}\right)\right]\cdot A_{i}$$

where $p_{i}^{\pm }$ is the probability that the *i*th surface patch, with area $A_{i}$, will have a shape-specific positive or negative value of S. The constants $S_{0}$, *a*, *b*, and *c* can be temperature-dependent, where $S_{0}$ has the dimension of entropy, and the rest has the dimension of entropy per unit area. In Equation (9) the area of a surface patch is multiplied by the constant *a*, only if it has an S value close to 1 (>0.99 in our implementation). Otherwise, the area of the surface patch is multiplied by *b* or *c*, depending on their deformations. The deformations, i.e., the deviation of shape index value from 1, arise when the surfaces of more than one atom overlap. As deformation reduces the MI of the system, we expect the overall signs of the terms related to the deformations to be negative. The individual plogp terms are all negative, and the constants *b* and *c* should be positive to make the deformation terms in our equation negative.

Given that all the other macroscopic conditions (pressure, temperature, and number of particles) are constant, the gas-phase entropy of different molecules can reflect non-additivity, i.e., follow an area law, as the molecules’ complexity and sizes increase. Gas-phase entropy is expressed as the molar entropy or entropy content of one mole of pure gas at standard pressure and temperature. Gas-phase entropy can be measured by measuring a gas’s heat capacity as a function of temperature. Please see [6] for a review of the experimental methods for measuring the thermodynamic properties of organic substances. To test the area law, we collected experimental values of the gas-phase entropy of 1942 small molecules. We fit the data to derive the constants $S_{0}$, *a*, *b*, and *c* and compared the results with the entropy calculation with molecular mechanics and quantum mechanics methods based on the rigid rotor harmonic oscillators (RRHO) approximation, which is explained in more detail later in the article, and compare the results. Ideally, the constants should be derived from an ab initio model of the area law of the molecular entropy. As the collection of molecules increases, the constants from fitting the experimental data should approach the universal constants.

To compare the dependency of entropy on molecular volume vs. area, we modeled gas-phase entropy as a function of molecular volume and area. The root mean square error (RMSE) in estimating gas-phase entropy from a simple area law, without any correction due to the deformation, was 27.98 J/mol·K (Table 1), vs. 35.96 J/mol·K from a similar volume-dependent law—reinforcing the notion that for a single molecule, where different parts of molecules have correlated dynamics, thermodynamic entropy varies as an area of the molecule rather than the volume.

## 2. Materials and Methods

### 2.1. Data Curation

As the first step, we built a database with experimental gas-phase entropy values (at 25 °C in J/mol·K) for various organic compounds (involving elements C, H, N, O, S, P, Cl, Br and I) curated from literature [25,26,27,28,29]. Overall, 1942 compounds with corresponding experimental entropies in the range 190–1040 J/mol·K were obtained (see Figure S1 in the Supplementary Materials). Most of the compounds (≈84%) had entropies below 500 J/mol·K (Figure S1 in Supplementary Materials) and had less than five rotatable bonds. High entropies (>700 J/mol·K) were associated with compounds containing more than 45 heavy atoms and more than ten rotatable bonds.

### 2.2. Molecular Structure Calculation

Our curated database contains SMILES strings of chemical compounds. The SMILES strings for each molecule were converted into a single set of 3D coordinates using RDKit (Release 2020.09.1.0) [30]. The atoms’ van der Waals (VDW) radii were assigned using the software OpenBabel (version 3.1) [31]. Note that the VDW assignment by OpenBabel does not depend on the local environment. For example, all carbon atoms will have the same VDW radius; no further parameterization is needed for individual small molecules. The structures were minimized using the Universal Force Field [32] (UFF) implemented in RDKit.

To understand the impact of conformational variability on $S_{\mathrm{th}}^{\mathrm{area}}$, we generated multiple conformers using RDKIT [33]. For some 30 randomly selected molecules (with more than three rotatable bonds), up to ten conformers were generated using RDKIT. Since the generated conformers can be structurally similar, only conformations that are at least a 0.50 Å root mean square deviation (RMSD) apart from one another were retained. As a result of this filtering, the final tally of generated conformers was less than 10 for some compounds. Each conformation was then subjected to geometry optimization using the AM1 [34] Hamiltonian in the semi-empirical program MOPAC [35].

### 2.3. Theoretical Entropy Calculation

To calculate the gas phase entropies for the molecules, the quantum-chemical Gaussian-4 [36] (G4) level of theory was used. The G4 theory has been shown to provide a good compromise for thermochemistry calculations in comparison to the other methods tested (see Ghahremanpour et al. [26] and Ghahremanpour et al. [37]). The G4 entropies for almost 1000 compounds were taken from the Alexandria library [37]. Entropies for an additional ∼500 compounds were computed in-house. Compounds with convergence issues and those that took more than 24 h of computing time were excluded. The calculations were performed using Gaussian 09 [38]. The OpenBabel tool *obthermo* was subsequently used to extract thermochemistry data from the Gaussian output files.

We also calculated entropy values estimated using normal-mode analysis, *S*_RRHO-NMA_, for 1665 molecules. The force field parameters for all the small molecules reported here were generated using the Open Force Field Toolkit (OPFF) [39]. The OPFF uses GAFF [40], AmberTools [41], and Antechamber [42] to generate the parameters. The structures of all compounds were first energy-minimized using GROMACS [43]. Normal mode analysis was then carried out using GROMACS, and the entropy estimates were obtained using the “gmx nmeig” module.

### 2.4. Molecular Surface Calculation

In this study, we approximate the electron density at the surface of an atom by a parametric exponential equation [44,45]:

(10) $$G\left(x\right)=\sum_{i}\exp\left(-\frac{\left(|x-r_{i}|-a_{i}\right)}{\sigma }\right)$$

where $r_{i}$ is the position of the *i*th atom centre and $a_{i}$ the corresponding VDW radius. An isosurface *M* can be defined as a collection of all x for which G(x) has a specific value, i.e., $M=G\left(c\right)\forall x\in R^{3}|G\left(x\right)=c$. However, such a surface may not be well defined; i.e., ∇G(x) must be defined for every x and should not be zero [46]. To have a well-defined surface function, the isosurface is rather defined as a collection of points where the inverse of the electron density function, $G^{-1}\left(x\right)$, has a specific value, i.e., $M=G^{-1}\left(c\right)\forall x\in R^{3}|G^{-1}\left(x\right)=c$ [46]. The adjustable smoothing parameter 0≤σ≤1 affects the level of detail associated with the surface. For instance, larger values of σ (typically >0.5) smooth out the surface details; i.e., cavities and protrusions are less visible, while smaller values of σ reveal more details of the surface. Please see Figure S3 in the Supplementary Materials to compare the impact of different σ on the level of surface detail. The isovalue *c* controls the volume enclosed by the surface. In this article, we calculated the surfaces at the isovalue c=1.0 [47] and used a smoothing factor (σ) of 0.1. The molecular surfaces were generated using the Marching cubes algorithm [48], using in-house software written in C++.

### 2.5. Surface Curvature

The VDW surface for the molecules defined by Equation (10) is a continuously differentiable function and is defined analytically at every point. From the analytical expression of the surface, we can calculate principal curvatures at every point. If we draw a small normal plane at a point on the surface and calculate the curvature of every line going through the point, then the highest and lowest curvatures of those lines, calculated at the point, are the principal curvatures $\kappa_{1}$ and $\kappa_{2}$ ($\kappa_{1}>\kappa_{2}$) of the surface at that point. The principal curvatures measure how the surface bends in different directions at a point (see do Carmo [49]) and can be calculated analytically from the first- and second-order partial derivatives of the surface function [50,51]. The principal curvatures can be further combined to define the shape index (S) [52]:

(11) $$\begin{array}{cc} S & =\frac{2}{\pi }\arctan\frac{\kappa_{1}+\kappa_{2}}{\kappa_{1}-\kappa_{2}}. \end{array}$$

The shape index (S) measures the local shape of a surface and varies from −1 (concave) to 1 (convex).

### 2.6. Shape Index Probabilities

The molecular surface, once calculated, is discretized by a triangle mesh using the Marching cubes algorithm, i.e., covering the surface with triangles. After triangulation, the shape index values are calculated at the center of each mesh triangle. The probabilities $p^{\pm }$ that a specific surface patch of a molecule will have a shape index value of $S^{\pm }$ are determined by binning the shape index values.

Thus, for a molecular surface defined by *k* triangles,

1. Calculate the shape index (see Equation (11)) at the center of each triangle as the mean of shape index values at the vertices. Divide the data into positive 0≥S<0.99 and negative groups (<0).
2. Calculate the normalized histogram counts for shape index distributions with respect to a predefined number of bins. The probabilities for each bin are given by $p_{i}=\frac{k_{i}}{k}$, where $k_{i}$ is the number of triangles (i.e., shape index values) allotted to the *i*th bin.

We should emphasize that we calculate each molecule’s $p^{\pm }$ values independently. The entropy calculation using Equation (9) can be impacted by the bin width and the smoothing factor of the parametric surface function. We varied the number of bins between 64, 128, and 256 and the smoothing factor σ between 0.1, 0.3, and 0.5. We then calculated the Pearson correlation coefficient between the shape entropies and the experimental gas-phase entropy to determine the effect of σ and the number of bins (see Table S1 in the Supplementary Materials). While the smoothing factor σ affects the correlations to some extent, the bin width or number has little to no impact. We chose 64 bins to calculate the histograms and the probability density using σ=0.1 for the rest of the work.

### 2.7. Data Fitting

To identify suitable values of the parameters (S0, a, b, c) in Equation (9), we used a genetic algorithm (GA). In the GA search, the upper and lower bounds for the parameters were set to ±200, ±100, ±50, ±50, while the population size was varied between 100 and 250. Crossover and mutation probabilities were set to 0.75 and 0.25, respectively. The fitness function to be maximized was set to the inverse of the RMSE and the algorithm ran for 100 cycles. The calculations were carried out using the GA [53,54] package in R (version 4.3.1).

## 3. Results and Discussion

From the literature, we curated 1942 experimental gas-phase entropy values of organic molecules, i.e., involving elements C, H, N, O, S, P, Cl, Br, and I. For the curated 1942 molecules, most of the $S_{\mathrm{Expt}}$ values were between 250 and 500 J/mol·K, and entropies typically increased with the number of atoms in the molecule (Figure S1 in Supplementary Materials). We calculated the structures of the 1942 molecules from their SMILES strings, and from the structures, we calculated the molecular surfaces and shape index values. Please see the Methods section for details of each of the steps. In subsequent sections, we show that the thermodynamical entropy calculated from a molecule’s area and shape index values has a collinear relationship with the gas-phase entropy. From the collinearity, we can estimate the gas phase of a molecule in a matter of seconds compared to tens to hundreds of CPU hours for other methods.

### 3.1. Gas-Phase Entropy Varies Linearly with Shape Entropy

**Figure 1:**
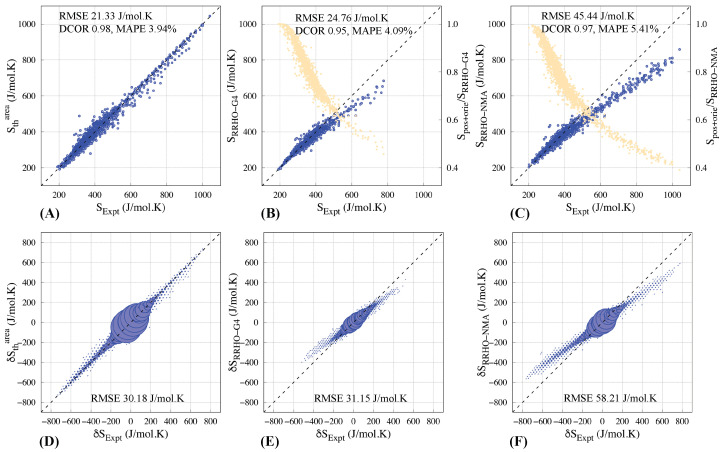
(**A**) $S_{\mathrm{th}}^{\mathrm{area}}$ values, the thermodynamical entropies calculated from the area law (Equation (9)), are plotted against experimental gas-phase entropies for 1942 molecules. The root mean square error (RMSE) between the calculated and experimental entropy is 21.34 J/mol·K. The correlation (DCOR) between the values, calculated using distance correlation (see Section 2), is 0.97, and the mean average percentage error (MAPE) is 3.94%. The dotted line represents the line where the values of the X and Y axes are equal. (**B**) Thermodynamic entropies were calculated using G4 quantum chemical calculations with the SHM approximation and plotted against experimental gas-phase entropies for 1529 molecules (blue dots). For the remaining 413 molecules, mainly the larger molecules, the G4 calculations did not converge. The orange dots represent the positional and orientational entropy as a fraction of the calculated total entropy. The error in the calculated entropy increases as the positional and orientational entropy falls below 60% of the calculated total entropy. (**C**) Thermodynamic entropies were calculated using normal mode analysis (NMA) and plotted against experimental gas-phase entropies for 1665 molecules (blue dots). The parameters could not be generated for the remaining 277 molecules (see Methods). The orange dots represent the positional and orientational entropy as a fraction of the calculated total entropy. (**D**) The differences in calculated (Y-axis) and experimental (X-axis) entropy of all possible pairs of molecules are plotted as histograms. The area of the circles is proportional to the number of molecule pairs the circle represents. Plots (**E**,**F**) represent the entropy differences calculated using G4 and NMA, respectively.

In our dataset, the thermodynamical entropy $S_{\mathrm{th}}^{\mathrm{area}}$ calculated from the surface features of a molecule showed a linear relationship with experimental gas-phase entropy, $S_{\mathrm{Expt}}$ (Figure 1), with $R^{2}$ values of ≈0.98 (Table 2). Note that, in this article, the area is measured in Å^2^. The RMSE with the experimental values is 21.32 J/mol·K, and the mean average percentage error (MAPE) is 3.94%. The *b* and *c* in Equation (9), the coefficients for the deformations, are, as expected, positive and are 6.910 and 21.456, respectively. The deformations indicate atomic bonds and consequently reduce the MI about a molecular system’s microstates. The linear relationship holds for the full range of the experimental gas-phase entropy values: 190–1040 J/mol·K. Even if the effect of the deformation is not taken into account, and the entropy is modeled as proportional to the surface area, the RMSE with the experimental values is 27.97 J/mol·K, and the MAPE is 5.42% (Table 2). We calculated the 95% confidence intervals using bootstrapping [55] for each parameter. Here, we conducted random data sampling with replacement and refitted the GA models for each replicate. We calculated the confidence intervals for the parameters based on 100 bootstrap replicates of the data.

To investigate if different possible conformations of a molecule change the $S_{\mathrm{th}}^{\mathrm{area}}$ values, i.e., whether we need to include something similar to $S_{\mathrm{conf}}$ in $S_{\mathrm{vib}}$, we generated multiple conformations (∼10) of randomly selected 30 molecules from the dataset (see Section 2) with 4 to 19 rotatable bonds) and calculated the $S_{\mathrm{th}}^{\mathrm{area}}$ for each of the conformations. The variation in $S_{\mathrm{th}}^{\mathrm{area}}$ between different conformations was less than 1% (ranging from ≈0.12% for molecules with 9 rotatable bonds to ≈0.68% for those with 16 rotatable bonds) for all the 30 molecules analysed. Consequently, in our dataset of small molecules, we concluded that $S_{\mathrm{th}}^{\mathrm{area}}$ is agnostic to the possible molecular conformations.

In this study, all surfaces were generated using a constant grid interval of 0.075 Å, which offers a good balance between the coarseness of the tessellation and the calculation time. To study the effect of the coarseness on *a*, we increased the interval to values of 0.09 Å and 0.125 Å for some randomly chosen molecules with 20 to 60 atoms. The increase in coarseness increased the surface areas by 0.1% and 0.2%, respectively. Given the marginal change in the overall surface area, we expect the impact on the value of *a* to be negligible.

### 3.2. The Impact of Positive and Negative Curvature on the Entropy

**Figure 2:**
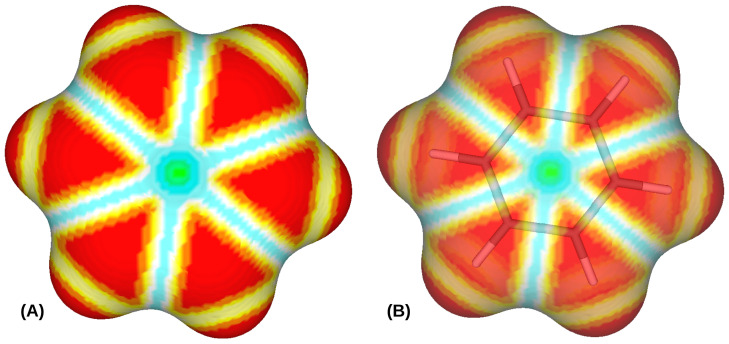
(**A**) shows the shape index (see Equation (11)) mapped to the molecular surface of benzene for σ=0.1. Shape index scales (ranging from −1 to 1) are divided into 9 categories as defined by Koenderink and van Doorn [52]: (i) $S\in [-1,-\frac{7}{8})$ shown in green, (ii) $S\in [-\frac{7}{8},-\frac{5}{8})$ in cyan, (iii) $S\in [-\frac{5}{8},-\frac{3}{8})$ in blue, (iv) $S\in [-\frac{3}{8},-\frac{1}{8})$ in pale blue (v) $S\in [-\frac{1}{8},\frac{1}{8})$ in white, (vi) $S\in [\frac{1}{8},\frac{3}{8})$ in pale yellow, (vii) $S\in [\frac{3}{8},\frac{5}{8})$ in yellow, (viii) $S\in [\frac{5}{8},\frac{7}{8})$ in orange, and (ix) $S\in [\frac{7}{8},1)$ in red. (**B**) shows a transparent version of (**A**), along with the embedded model of benzene represented by sticks.

The value of the shape index (S), our definition of surface curvature, for a perfectly spherical surface is 1, and an inverted concave spherical surface is −1. As surface features move from convex to concave, the S value goes from 1 to −1. S=0 represents a saddle, where these two different, positive and negative, curvature meets. In our surface representations of molecules, 0<S<1 ($S^{+}$) appears near the surface where two atoms form a bond, whereas −1<S<0 ($S^{-}$) appears where more than two atoms, i.e., more than one patch with $S^{+}$, overlap. For example, in benzene, $S^{+}$ is at the C-H bonds, and $S^{-}$ is at the intersection of two C-H bonds and the center of the carbon ring (Figure 2). As expected, contributions from the deformation terms are negative. Note that contributions from plog(p) terms in Equation (9) are all negative, and the coefficients *b* and *c* are positive, making the contributions from the deformation terms negative, i.e., reducing the entropy. In our model, atomic surfaces are spherical, and deformations or any deviation from the sphericity indicates bonds between the atoms. Any bond between two atoms represents constraints in their relative movements and vibrations. Consequently, a system with two bonded atoms will have reduced MI compared to a system with two free atoms. Moreover, the absolute coefficient value of the term corresponding to $S^{-}$ in Equation (9) (21.46—Table 2), is substantially higher than that of $S^{+}$ (6.91—Table 2), as $S^{+}$ represents the presence of bonds between two atoms, whereas $S^{-}$ represents the presence of bonds between multiple and more than two atoms, thus reducing our MI of the system to a great extent.

In our datasets, the major fraction of surfaces have an S value between 0 and 0.99 (61.7%). Another 23.9% of the surface has S values > 0.99, and the remaining 14.4% of the surface has S values < 0 (Figure S2 in Supplementary Materials).

### 3.3. The Coefficient of Ultraviolet Cutoff—Connecting $S_{BH}$, $S_{entanglement}$, and $S_{th}^{area}$

The ultraviolet cutoff, the minimal value of the length used in the derivation of the area law, in Equation (6) is the $L_{P}$, the Planck length. The exact definition of the ultraviolet cutoff, $L_{UV}$, will depend on the system under study; for example, if the system under study is a crystal, then $L_{UV}$ would be the atomic spacing. The coefficient for the ultraviolet cutoff, $C_{\mathrm{UV}}$, in Equation (6) is $\frac{1}{4}$. For a system with *N* coupled harmonic oscillators, Srednicki showed that the $C_{\mathrm{UV}}$ is 0.30 [19], very close to $\frac{1}{4}$. To calculate the $C_{\mathrm{UV}}$ in our system, we should change the entropy values from J/mol·K for one mole of molecules to J/K for one molecule. The ultraviolet cutoff for our derivation is 1.1 Å, the VDW radius of the hydrogen atom we used. If we ignore the constant term in Equation (9) and focus on the coefficient of the term proportional to the area, we can write

(12) $$\begin{array}{cc} \frac{S_{\mathrm{th}}^{\mathrm{area}}\;\mathrm{in}\;J/\mathrm{mol}\cdot K}{A\;\mathrm{in}\;{\text{Å}}^{2}} & ∼a\\ \frac{S_{\mathrm{th}}^{\mathrm{area}}\;\mathrm{in}\;J/K}{A\;\mathrm{in}\;{\text{Å}}^{2}} & ∼\frac{a}{N_{a}}\\ \mathrm{Using}\;\;\;\mathrm{Equation}\;(6) & \\ C_{\mathrm{UV}}\frac{N_{a}k_{B}\;\mathrm{in}\;J/K}{{\left(L_{UV}\;\mathrm{in}\;\text{Å}\right)}^{2}} & ∼a\\ \frac{a{\left(L_{UV}\;\mathrm{in}\;\text{Å}\right)}^{2}}{N_{a}k_{B}\;\mathrm{in}\;J/K} & ∼C_{\mathrm{UV}} \end{array}$$

where *A* is the area and $N_{a}$ is Avogadro’s number. The constant *a* is 3.078 in Equation (9) ($S_{\mathrm{th}}^{\mathrm{area}}$ in Table 2, row 1), and 1.948 J/K·Å^2^ if we ignore the deformation terms ($S_{\mathrm{area}}$ in Table 2, row 2). We included $S_{area}$ in this analysis of $C_{UV}$ to compare them with the $C_{UV}$ values in black hole entropy and quantum entanglement entropy for N coupled oscillators, where no deformation terms were used. Putting $L_{UV}=1.1$ Å, and the *a* values in Equation (12), we obtain $C_{\mathrm{UV}}$ 0.41 and 0.26, respectively—values very close to the $C_{\mathrm{UV}}$ values in black hole entropy and quantum entanglement entropy for *N* coupled harmonic oscillators. Interestingly, for the model where deformation terms are ignored ($S_{\mathrm{area}}$), the $C_{\mathrm{UV}}$ is 0.26, or $\frac{1}{3.9}$, tantalizingly close to the $\frac{1}{4}$ term in black hole entropy, where the wrinkles in the black hole surface are also ignored. Note that the power of $S_{\mathrm{area}}$ model to predict gas-phase entropy is at par with or better than the other currently popular methods compared in this article (Table 2 and Figure S5 in Supplementary Materials). The area law of entropy, calculated and measured in three different types of complex systems of three vastly different physical dimensions, returns a constant with similar values on all three occasions, thus validating the pertinent idea behind the theory.

### 3.4. Comparison with Entropies Calculated Using RRHO Approximation

The RRHO model, which approximates a molecule as a collection of harmonic oscillators, is the most commonly used method to calculate the entropy of a molecule. We curated and calculated entropies, *S*_RRHO-G4_, for 1529 molecules using the quantum-chemical Gaussian-4 (G4) theory (see Methods). We also calculated entropies, *S*_RRHO-NMA_, for 1665 molecules using NMA and molecular mechanics forcefields (see Methods). Both methods show collinearity with the experimental gas-phase entropies for smaller molecules with values less than ≈500 J/mol·K (Figure 1B,C). For larger molecules, the methods start deviating from the collinearity (Figure S4 in Supplementary Materials). RRHO methods use analytical formulas to calculate the positional and orientational entropy ($S_{\mathrm{RR}}$). For gas-phase entropy less than ≈500 J/mol·K, $S_{\mathrm{RR}}$ consists of a significant fraction of the entropy values. As the $S_{\mathrm{RR}}$ falls below 60% of the total entropy, the deviation from the collinear behavior becomes apparent (Figure 1B,C). A possible reason can be that contributions from $S_{\mathrm{anharm}}$ and $S_{\mathrm{conf}}$ in $S_{\mathrm{vib}}$, which the harmonic oscillator approximation cannot capture, increase as the size of a molecule increases. Note that a recent study identifies that conformational entropy accounts for ≈<5% of gas-phase entropy in small molecules [10]. Another possible reason is that the $S_{\mathrm{orie}}$ is not decoupled from $S_{\mathrm{vib}}$ for larger molecules, as approximated in the RRHO model.

*S*_RRHO-G4_ and *S*_RRHO-NMA_ values have RMSE values of 24.77 and 45.44 J/mol·K, respectively, with $S_{\mathrm{Expt}}$ (Table 2). The values for MAPE for *S*_RRHO-G4_ and *S*_RRHO-NMA_ are 4.09% and 5.41%, respectively (Table 2). The huge difference in RMSE values between *S*_RRHO-G4_ and *S*_RRHO-NMA_ is primarily because *S*_RRHO-G4_ contains the values for smaller molecules, where $S_{\mathrm{RR}}$ dominates the entropy values. The calculation of *S*_RRHO-G4_ did not converge for larger molecules. Furthermore, in our dataset, the coefficient of determination, $R^{2}$, is higher between *S*_RRHO-NMA_ and $S_{\mathrm{Expt}}$, 0.97, than between *S*_RRHO-G4_ and $S_{\mathrm{Expt}}$, 0.95 (Table 2). If we include molecules present in all three different sets, $S_{\mathrm{th}}^{\mathrm{area}}$, *S*_RRHO-G4_, and *S*_RRHO-NMA_, and calculate RMSE based on those 1326 molecules, the RMSE values are 20.91, 23.38, and 26.21 J/mol·K, respectively (Table 1). Similarly, for the 1529 molecules common to $S_{\mathrm{th}}^{\mathrm{area}}$ and *S*_RRHO-G4_ sets, RMSE values are 21.47 and 24.76 J/mol·K, respectively (Table 1). And for the 1665 molecules common to $S_{\mathrm{th}}^{\mathrm{area}}$ and *S*_RRHO-NMA_ sets, RMSE values are 20.93 and 45.44 J/mol·K, respectively (Table 1). As the size of the molecules increases, the power of $S_{\mathrm{th}}^{\mathrm{area}}$ to represent both $S_{\mathrm{RRHO}}$ and $S_{\mathrm{anharm}}$ becomes apparent.

### 3.5. Prediction of Relative Entropies

Often, relative entropy, i.e., the difference in entropy values between two molecules, is a more helpful quantity than the individual absolute entropy values. To compare the performances of three entropy models in predicting differences in experimental gas-phase entropy values, we calculated the experimental gas-phase entropy difference, $\delta S_{\mathrm{Expt}}$, between all possible pairs of the molecules in a dataset. We compared them with the corresponding difference in the modeled entropy values—$\delta S_{\mathrm{th}}^{\mathrm{area}}$, *δS*_RRHO-G4_, and *δS*_RRHO-NMA_. The RMSEs between the relative entropy values are 30.18, 31.15, and 58.21 J/mol·K for $\delta S_{\mathrm{th}}^{\mathrm{area}}$, *δS*_RRHO-G4_, and *δS*_RRHO-NMA_, respectively.

## 4. Conclusions

Entropy encapsulates missing information (MI) or our ignorance about a system. After decades of theoretical works, since the Bekenstein–Hawkins work on black hole entropy, physicists are converging on the idea that the area of the horizon describes our ignorance or the MI of the matter that has fallen in—all different ways of internally arranging the building blocks of the black hole to match its outward appearance without knowing what the microstates are. Similarly, for a gas made of molecules, we know the temperature—the average speed of particles—but not what every particle is doing, and the gas’s entropy reflects the MI about the number of ways the particles can organize themselves. For two different physical systems of different dimensions, black holes, and the entanglement of quantum particles, the area of the systems is proportional to the MI or entropy. We show that proportionality, or the area law, also holds for the thermodynamic entropy of gaseous molecules. The coefficient in the area law in our gas-phase entropy is very close to those in the laws of entropy in the black hole and quantum entanglement area, indicating the robustness of the underlying idea in three systems of vastly different dimensions.

Different conformations of the molecules in our dataset had less than 1% variation of areas between them. Consequently, considering a single conformation for the calculation of the entropy using area-law was accurate enough for the dataset. For larger flexible molecules, such as peptides and other biomolecules, their flexibility may require the explicit calculation of different stable conformations of the molecules. However, the experimental entropy for most biomolecules is available in solution. Currently, the application of the method is limited to the gas-phase entropy of small molecules. The current model needs to be modified to incorporate the effect of solvation to create a semi-empirical model for the solvation entropy of larger molecules and compare the performance with the experimental results.

Calculating thermodynamical entropy using the area law allows for calculating molecular entropy faster and more accurately than the currently popular way of approximating the molecules as a collection of harmonic oscillators. In our model, we approximated the atoms to have spherical surfaces with VDW radii. Furthermore, each type of element has been assigned a single VDW value. In molecular mechanics force fields, used in the calculations of *S*_RRHO-NMA_, the VDW radii depend on the element type and the atom’s local environment. Consequently, the same element can have different VDW radii in different atomic environments. The definition of atomic surface and the VDW radii, possibly considering the atomic environment, can be updated to improve the accuracy of the area law in our model. The speed and accuracy of our method will open up new possibilities for the explicit inclusion of entropy in computational biology methods, such as molecular docking or QSAR (quantitative structure–activity relationships) methods and other methods related to virtual screening.

## Figures and Tables

**Table 1:** RMSE reported for different combinations based on the data common to the $S_{\mathrm{th}}^{\mathrm{area}}$, *S*_RRHO-G4_ and *S*_RRHO-NMA_ datasets. Numbers in brackets indicate the number of compounds in common for the groups considered.

	$S_{\mathrm{th}}^{\mathrm{area}}$	***S*_RRHO-G4_**	***S*_RRHO-NMA_**
$S_{\mathrm{th}}^{\mathrm{area}}$ ∩ *S*_RRHO-G4_ ∩ *S*_RRHO-NMA_ (1326)	20.91	23.38	26.21
$S_{\mathrm{th}}^{\mathrm{area}}$ ∩ *S*_RRHO-NMA_ (1665)	20.93	–	45.44
$S_{\mathrm{th}}^{\mathrm{area}}$ ∩ *S*_RRHO-G4_ (1529)	21.47	24.76	–

**Table 2:** Root mean square error (RMSE), coefficient of determination ($R^{2}$), and mean average percentage error (MAPE values) between predicted and experimental gas-phase entropy. In $S_{\mathrm{area}}$, we assumed the entropy is proportional to the surface area and ignored any contribution from the deformations in the surface. In this article, the area is measured in Å^2^. In $S_{\mathrm{th}}^{\mathrm{area}}$, we added the contribution of the deformations. In G4 and NMA, entropy values were calculated using rigid rotor harmonic oscillator (RRHO) approximation, where the vibrational entropies were calculated using density functional theory and normal mode analysis (NMA) using molecular mechanics forcefield, respectively. We calculated $S_{\mathrm{area}}$ and $S_{\mathrm{th}}^{\mathrm{area}}$ for all 1942 molecules, and the only parameters used were the atomic Van Der Waals radii of the atoms from OpenBabel [31], which calculates a single VDW radius for each element regardless of its environment using the method described in [56]. For G4, we curated and calculated the entropies for 1529 molecules. The calculations for the rest of the molecules did not converge in our stipulated time frame. For NMA, we calculated entropies for 1665 molecules. We could not confidently generate the forcefield parameters for the rest of the molecules. We used bootstrap methods (100 bootstraps) to calculate lower and upper limits of the 95% confidence interval. Upper and lower limits are shown as raised and lowered numbers, respectively.

Method	$S_{\mathrm{gasphase}}$ = (J/mol·K)	RMSE (J/mol·K)	$R^{2}$	MAPE %
Area	$S_{\mathrm{area}}=118.84_{118.62}^{119.23}+\sum_{i}1.948_{1.945}^{1.959}\;A_{i}$	$27.977_{27.841}^{28.112}$	$0.957_{0.955}^{0.958}$	5.42
Area + deformation	$S_{\mathrm{th}}^{\mathrm{area}}=104.32_{104.06}^{104.47}+$ $\sum_{i}[3.078_{3.068}^{3.083}+6.910_{6.819}^{6.980}\;p_{i}^{+}\log\left(p_{i}^{+}\right)$ $+21.456_{21.352}^{21.726}\;p_{i}^{-}\log\left(p_{i}^{-}\right)]\cdot A_{i}$	$21.326_{21.281}^{21.542}$	$0.975_{0.974}^{0.976}$	3.94
G4 NMA	$S_{\mathrm{RRHO}}=S_{\mathrm{pos}}+S_{\mathrm{orie}}+S_{\mathrm{HO}}$	$24.762_{23.180}^{26.480}$ $45.441_{42.86}^{48.51}$	$0.945_{0.937}^{0.953}$ $0.971_{0.976}^{0.967}$	4.09 5.41

## Data Availability

All data used in this study are available in the Supporting Information. The gas phase entropy prediction is available online at https://vvishwesh.github.io/surfent.

## Supplementary Materials

The following supporting information can be downloaded at: https://www.mdpi.com/article/10.3390/e26080688/s1. An Excel file with the observed and predicted gas-phase entropies for the different compounds (in SMILES format), including the gas-phase entropy calculated using NMA with the molecular mechanics force fields GAFF-ESP and GAFF-BCC. A brief introduction to von Neumann entropy and quantum entanglement entropy. Figure S1, showing the statistics of curated data, and Figure S2 shows the distribution of shape index values S in our dataset. Figure S3, showing the effects of smoothing parameter σ, and Table S1, showing the effect of the number of bins for calculating the histogram on the shape entropy. Figure S4, showing the performance of *S*_RRHO-G4_ and *S*_RRHO-NMA_ as a function of number of atoms, and Figure S5, showing the performance of $S_{\mathrm{area}}$ in predicting $S_{\mathrm{Expt}}$. Reference [57] is cited in the Supplementary materials.

## Abbreviations

The following abbreviations are used in this manuscript:

CPU	Central Processing Unit
DCOR	Distance Correlation
GA	Genetic Algorithm
LIGO	Laser Interferometer Gravitational-wave Observatory
MAPE	Mean Average Percentage Error
MI	Missing Information
NMA	Normal Mode Analysis
OPFF	Open Force Field Toolkit
RMSD	Root Mean Square Deviation
RMSE	Root Mean Square Error
RRHO	Rigid Rotor Harmonic Oscillators
SHM	Simple Harmonic Oscillators
UFF	Universal Force Field
VDW	van der Waal
